# Dual Targeting of Multiple Myeloma Stem Cells and Myeloid-Derived Suppressor Cells for Treatment of Chemotherapy-Resistant Multiple Myeloma

**DOI:** 10.3389/fonc.2021.760382

**Published:** 2021-11-10

**Authors:** Fatih M. Uckun

**Affiliations:** ^1^ Department of Developmental Therapeutics, Immunology, and Integrative Medicine, Drug Discovery Institute, Ares Pharmaceuticals, St. Paul, MN, United States; ^2^ Clinical Research Program, Aptevo Therapeutics, Seattle, WA, United States; ^3^ Translational Oncology Program, Reven Pharmaceuticals, Westminster, CO, United States

**Keywords:** tumor microenvironment (TME), multiple myeloma (MM), bispecific T-cell engagers (BiTEs), bispecific antibodies (BsABs), bone marrow microenvironment (BMME), myeloid-derived suppressor cells (MDSC)

## Abstract

Here we review the insights and lessons learned from early clinical trials of T-cell engaging bispecific antibodies (BsABs) as a new class of biotherapeutic drug candidates with clinical impact potential for the treatment of multiple myeloma (MM). BsABs are capable of redirecting host T-cell cytotoxicity in an MHC-independent manner to malignant MM clones as well as immunosuppressive myeloid-derived suppressor cells (MDSC). T-cell engaging BsAB targeting the BCMA antigen may help delay disease progression in MM by destroying the MM cells. T-cell engaging BsAB targeting the CD38 antigen may help delay disease progression in MM by depleting both the malignant MM clones and the MDSC in the bone marrow microenvironment (BMME). BsABs may facilitate the development of a new therapeutic paradigm for achieving improved survival in MM by altering the immunosuppressive BMME. T-cell engaging BsiABs targeting the CD123 antigen may help delay disease progression in MM by depleting the MDSC in the BMME and destroying the MM stem cells that also carry the CD123 antigen on their surface.

## Multiple Myeloma and Drug Resistance

MM is a heterogenous hematologic malignancy and relapses due to resistant disease are common (1–4). Resistance of the malignant clones to multiple drugs hampers a more successful treatment outcome after contemporary standard of care regimens in MM (1–4). Personalized therapy platforms have been designed to overcome the drug resistance, including precision medicines, kinase inhibitors, CAR-T cells, and antibody therapeutics (5–8). Effective treatment of patients with drug-resistant relapsed disease continues to be an unmet medical need (1–4).

## Immunosuppressive Bone Marrow Microenvironment in Multiple Myeloma

The immunosuppressive bone marrow microenvironment (BMME) in MM contains cellular elements that facilitate the immune evasion of malignant MM clones (9–13). These immunosuppressive cells include MDSCs, an immature myeloid cell population capable of inhibiting effector cytotoxic T-cell (CTL) populations as well as natural killer (NK) cells and contribute to the T-cell exhaustion which is a hallmark of the BMME in MM patients (4, 14–20). In addition, regulatory T cells (Tregs), regulatory B-cells (Bregs), and tumor-associated macrophages (TAM) also contribute to BMME-associated immunosuppression (20). The immunosuppressive BMME in MM has been implicated in clonal evolution and immune evasion of MM cells accelerating disease progression (4, 20).

**Figure 1:**
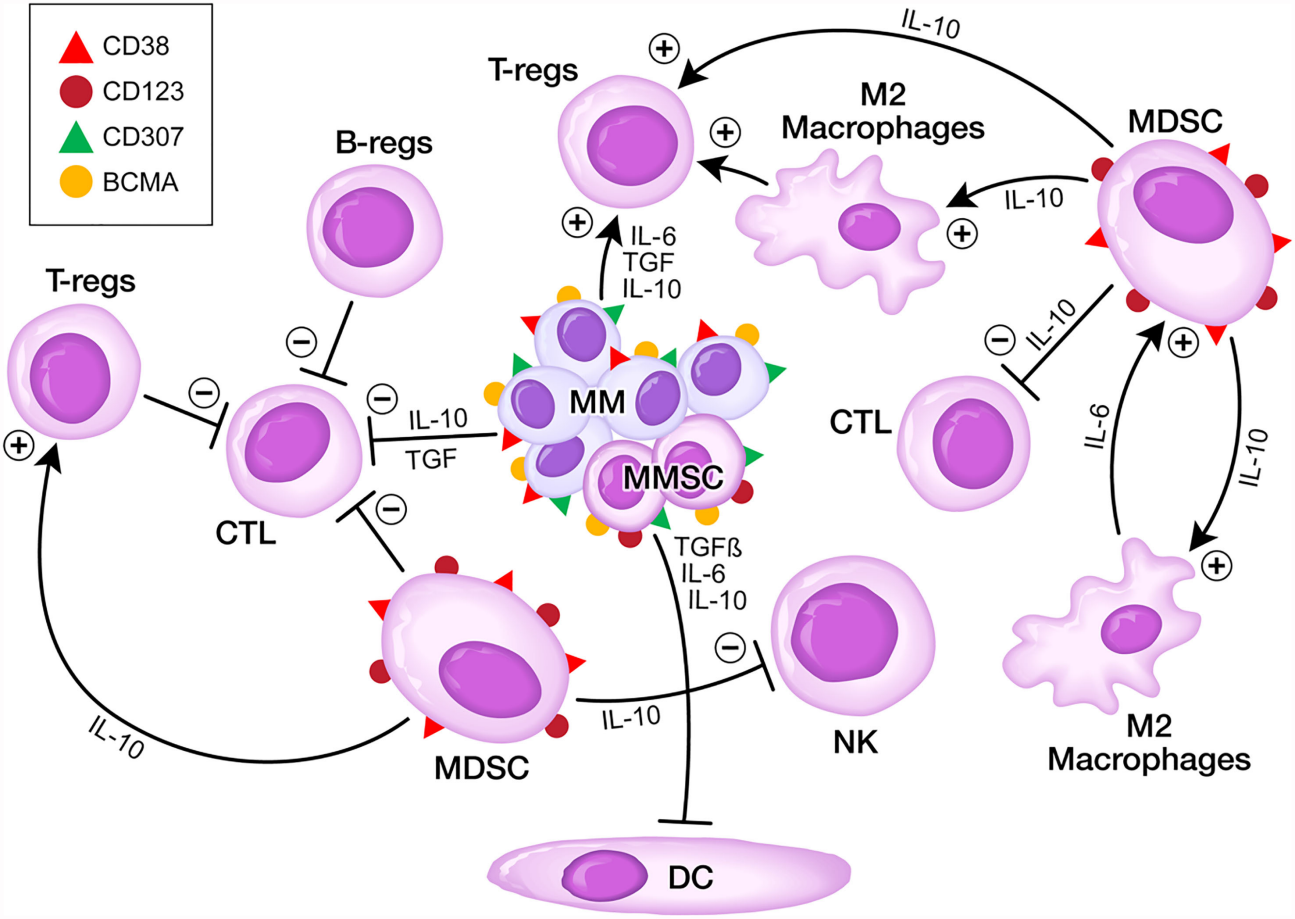
Targeting the Immunosuppressive TME in MM. MM stem cells (MMSC) express BCMA, CD38, CD307 and CD123 antigens on their surface. MDSCs express CD38 and CD123 antigens on their surface.

Expanded populations of MDSC, representing CD33^+^CD123^+^ immature myeloid cells within the bone marrow mononuclear cell fraction contribute to the immunosuppressive BMME by inhibiting both memory and cytotoxic effector T-cell populations as well as natural killer (NK) cells, thereby promoting the immune evasion of MM clones (4, 9–15, 20) (**Figure 1**). MDSCs along with MM cell derived interleukin 10 (IL-10), TGF-β and IL-6 inhibit dendritic cell (DC) maturation and their antigen-presenting function, which further aggravates the immunosuppression (6). The abundance of MDSCs is associated with a higher risk of rapidly progressive disease and poor survival outcomes in MM (9–11). MDSCs are activated by exosomes and support the development of Tregs, promote angiogenesis and growth of MM cells besides inhibiting the immune effector cells (21–26).

Several strategies are being explored to overcome the immunosuppressive cellular elements of the BMME in MM patients, including the use autologous hematopoietic stem cell transplantation (AHSCT) (4, 20, 27–29) to remodel the BMME by establishing a more favorable ratio between effective MM-specific CTLs *versus* Tregs and other immunosuppressive cells. Treatment strategies aimed at further enhancing the anti-MM immunity can be employed as post-AHSCT interventions, including MM- or MDSC-directed monoclonal antibodies (MoAb) (20, 27, 28). New generation multi-parameter minimal residual leukemia (MRD) detection techniques provide a unique opportunity to evaluate the effect of new treatment modalities that are applied as part of AHSCT or post-AHSCT on the quality and length of complete remission in both newly diagnosed high-risk MM as well as RR MM (29, 30).

## Dual Targeting of MM Cells and MDSCs

Dual targeting of MM cells and MDSCs using biotherapeutic agents has emerged as a very promising new therapeutic platform with a particularly high clinical impact potential. For example CD38 antigen is present on MM cells as well as MDSCs (4, 20, 31–34). Daratumumab, a complement-activating anti-CD38 MoAb capable of causing antibody‐dependent cellular cytotoxicity (ADCC) and apoptosis in MM cells, showed significant single agent activity in relapsed MM patients and improved the survival outcome when used in combination with other active anti-MM agents such as bortezomib and dexamethasone or lenalidomide and dexamethasone (4, 20, 31–34). Similar results were obtained using alternative anti-CD38 MoAbs, such as Isatuximab (4, 20). Daratumumab has been shown to expand the immunoreactive CTL populations *via* depletion of CD38^+^ immunosuppressive cellular elements of the BMME (33). The immunomodulatory effects of Daratumumab improved the clinical responses of previously resistant MM patients to standard combination therapy (4, 20). Unfortunately, increased expression levels of complement inhibitors CD55 and CD59 as well as decreased cell surface expression levels of CD38 on MM cells may decrease the clinical activity of anti-CD38 antibodies (4, 35).

## Clinical Impact Potential of Bispecific T-cell Engagers

**Figure 2:**
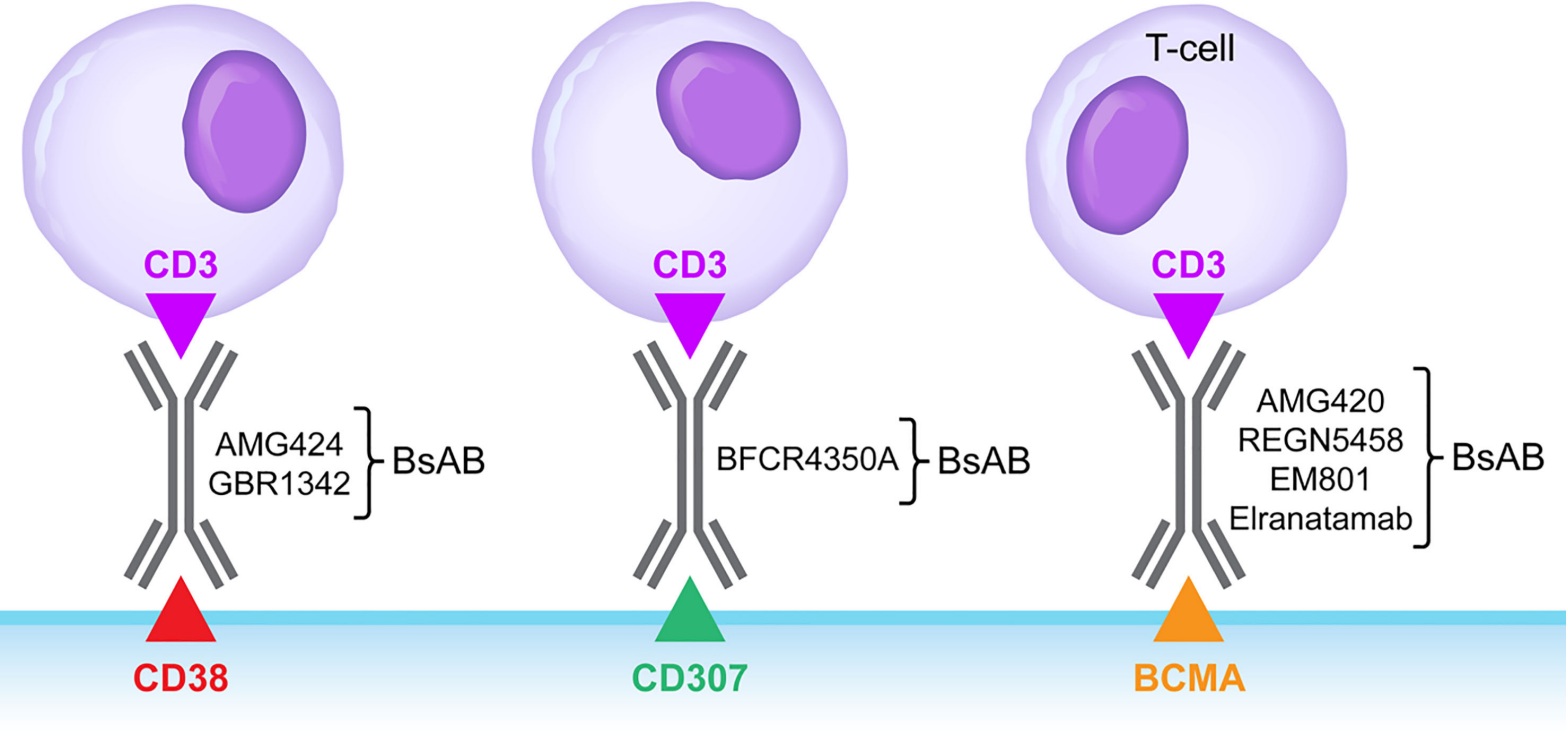
Bispecific Antibodies targeting MM cells. BsAB, bispecific antibody.

BsABs capable of redirecting host T-cell cytotoxicity in an MHC-independent manner to malignant clones as well as immunosuppressive MDSCs (14–20, 35–39) are being explored as a new class of drug candidates in various hematologic malignancies (40). Bispecific CD3xBCMA antibodies targeting the B-cell maturation antigen (BCMA; CD269/TNFRS17) on MM cells, such as EM801, REGN5458 (NCT03761108) and AMG 420 (NCT03836053) showed single agent activity in relapsed/refractory MM patients (41–46) (**Figure 2**). CD3xBCMA BsABs, Elranatamab (PF-06863135) and Teclistamab are being evaluated in R/R MM patients (NCT04649359 and NCT03145181/NCT04557098). In the MajesTEC-1 Phase 1 study of the BCMAxCD3 BsAB Teclistamab in R/R MM (NCT03145181), both intravenous and subcutaneous (s.c) administration schedules were evaluated, and the recommended phase 2 dose was identified as 1.5 mg/kg administered s.c. once a week. At this dose level, the overall response rate was 65%. Grade 3-4 neutropenia was observed in 40% and Grade 1-2 CRS was observed in 70% of the patients (47). The second-generation CD3xBCMA BsAB AMG701 that has an Fc domain to extend its half-life (48) is being evaluated in an Phase 1/2 clinical study (NCT03287908). TNB-383B has been designed to reduce the risk of the class-specific AE cytokine release syndrome (CRS) (49). TeneoBio has shown that TNB-383B causes significantly lower cytokine release from activated T-cells. A clinical proof of concept study (Clinicaltrials.gov identifier: NCT0302577) was designed to study the effects of reducing the levels of γ-secretase cleaved soluble BCMA in MM patients by using a γ-secretase inhibitor because soluble BCMA interferes with the mechanism of action of BCMA-targeting bispecific antibodies.

Bispecific CD3xCD38 antibodies have also been developed (50, 51) and entered clinical trials in patients with relapsed or refractory MM, such as AMG424 (NCT0344566) (51) and GBR1342 (NCT0330911). A CD38-reactive tri-specific antibody targeting CD3 and CD28 co-receptors on T-cells has also been developed to achieve augmented and sustained T-cell activation *via* CD28 engagement (52). A bispecific T-cell engaging CD3xCD307 antibody, named BFCR4350A, was developed targeting the FcRH5/CD307 antigen (53) on MM cells and it is currently being evaluated in a Phase 1 clinical trial (NCT03275103) (54).

Talquetamab is a GPRC5DxCD3 BsAB targeting the orphan G protein-coupled receptor GPRC5D that is abundantly expressed on MM cells. In a Phase 1 study in R/R MM patients testing both IV and SC administration schedules (NCT03399799), the RP2D was identified as 405 mcg/kg administered SC on a weekly basis (55). The RP2D level was well tolerated and exhibited promising activity with an overall response rate of 63%. CRS (79%), neutropenia (64%), anemia (57%) and dysgeusia (57%) were the most common treatment-emergent AEs. Furthermore, 7% of patients developed neurotoxicity and 32% developed infections. The overall response rate at the RP2D was 63% (55).

## Targeting CD123 on MDSC

The α-chain of the IL-3 receptor, also known as the CD123 antigen, is abundantly expressed on MDSC (20). Furthermore, CD123 is also expressed on plasmacytoid dendritic cells (PDCs) that contribute to the growth of MM cells as well as cancer stem-like cells and osteoclast progenitors (56). Several biotherapeutic agents targeting CD123 have been developed, including the CD123-directed recombinant human IL3 fusion toxin Tagraxofusb (SL-401), MoAbs, BsABs targeting CD123 antigen, such as bispecific T-cell engagers (BiTEs), dual-affinity retargeting antibodies (DARTs), bispecific killer cell engagers, and tri-specific killer cell engagers (40, 57–59).

Targeting the BMME in MM with SL-401 has been shown to reduce the viability of PDCs and inhibit PDC-induced MM cell growth, impair the viability of CD123^+^ MM stem cells, and prevent osteoclastogenesis in preclinical model systems (60). SL-401 is being assessed in combination with standard of care in a clinical study (NCT02661022) in relapsed/refractory MM patients with promising early evidence of clinical activity (61, 62). Seroproteomics analysis of MM patient serum samples reportedly showed a reduction of PDC-derived soluble proteins in SL-401 treated patients (63).

**Figure 3:**
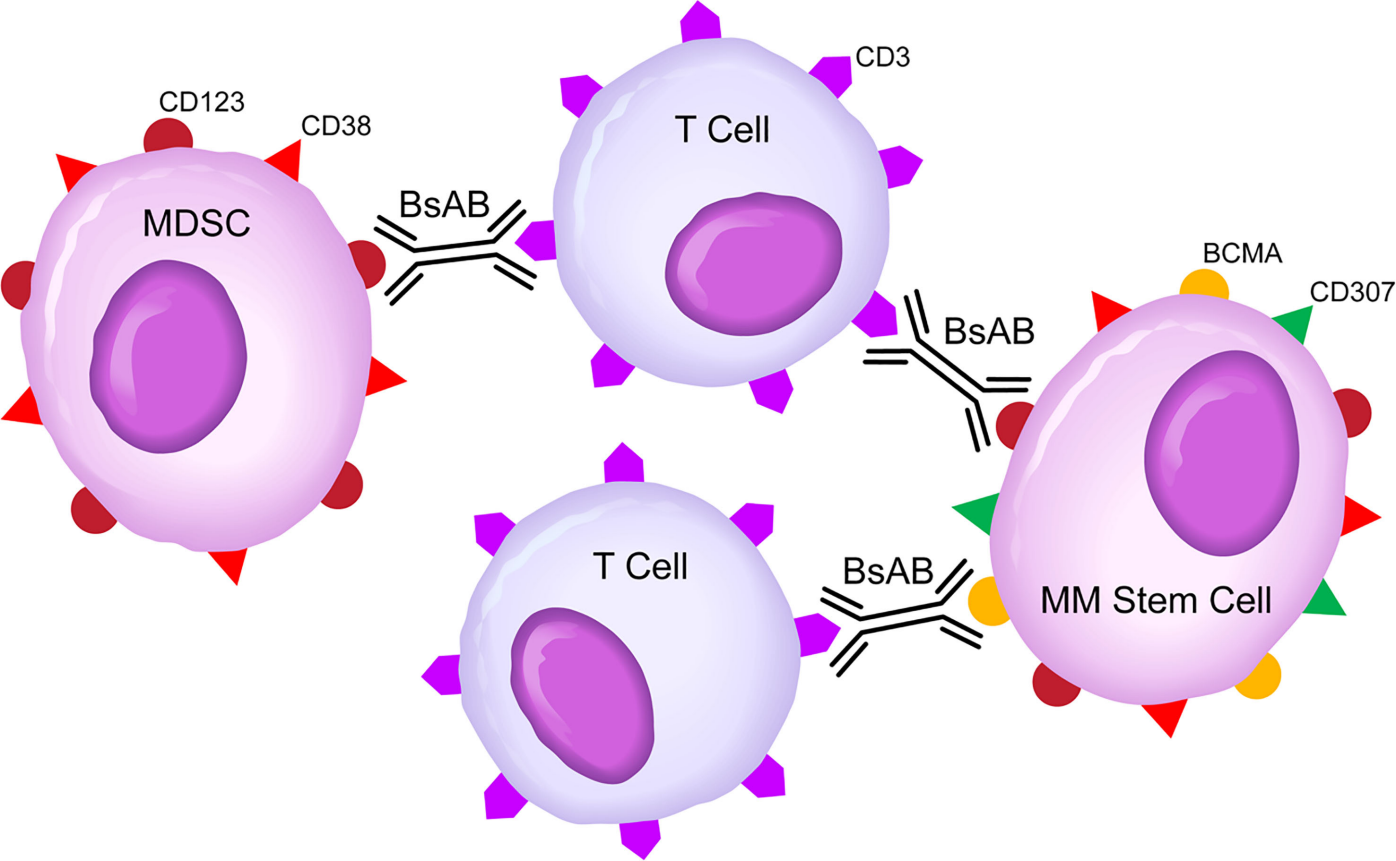
Bispecific CD3xCD123 Antibodies For Dual Targeting of MM Stem Cells Clones and MDSC Cells in High-Risk MDS Patients. BsAB, bispecific antibody; MM, Multiple myeloma; MDSC, Myeloid-derived suppressor cell. See text for a detailed discussion of the rationale of targeting the CD123 and CD38 antigens that are expressed on both MM stem cell clones and MDSCs.

CD123-targeting, CD3-engaging BsAB, such as Flotetuzumab (59) and APVO436 (64) bring cytotoxic T-cells (CTLs) within close vicinity of target CD123^+^ cells to create “cytolytic synapses” as a short bridge between target cells and CTLs, triggering CTL activation and destruction of targeted cells (**Figures 2**, **3**). These dual-function anti-MM drug candidates are currently in clinical trials for treatment of CD123-expressing hematologic malignancies with early clinical proof of concept for their ability to destroy CD123+ malignant clones, including CRs in relapsed or refractory AML patients (NCT02152956, NCT03647800). However, the clinical potential of a CD123xCD3 bispecific antibody in MM therapy may be limited as the bulk population of MM cells lack CD123 and depletion of CD123^+^ MM stem cells alone is unlikely to be an effective strategy for monotherapy. Therefore, clinical feasibility and efficacy studies of combinations of CD123 targeting BsAB with active anti-MM drugs such as pomalidomide that appeared to have augmented activity in the presence of the anti-CD123 fusion toxin tagraxofusp (3, 4, 8, 60), biotherapeutic agents, such as CD3xBCMA BsABs, daratumumab, elotuzumab (4–7, 20), and CAR-T cells (4, 65) are needed to gain insights into the clinical impact potential of CD123xCD3 BsABs.

Notably, MDSCs have been shown to significantly suppress the CTL engaging activity of a BCMAxCD3 BsAB, but the MDSC-suppressed CTL activity could be restored by addition of a hypomethylating agent (HMA) capable of epigenetically altering the MDSC transcriptome *via* reversal of the aberrant DNA methylation (66). Therefore, MDSC-targeting BsAB could be potentiated by HMAs. It is noteworthy that a combination of the CD123xCD3 BsAB APVO436 with azacitidine is being evaluated in one of the cohorts in the ongoing expansion phase of a Phase 1B AML study (NCT03647800).

## Cytokine Release Syndrome (CRS)

CD3-engaging BsABs act as agonists and activate T-cells in the presence of tumor cells expressing the target tumor-associated antigen, which can lead to excessive T-cell activation with release of inflammatory cytokines and development of the potentially life-threatening systemic inflammation, known as cytokine release syndrome (CRS) (67–71). For example, BsAB AMG330 binds CD33 antigen on AML cells as well as MDSCs cells and CD3ɛ on T-cells. In an open-label Phase 1 study (Clinicaltrial.gov identifier: NCT#02520427), AMG330 was given at doses ranging from 0.5–720 μg/d in the manner of continuous IV infusion among 55 patients with R/R AML (NCT02520427). AMG 330-related AEs included CRS (67%; Grade ≥3 in 13%) as the most frequent AEs (72). Similarly, CRS was observed in 63% of AML patients treated with AMG673, a new version of AMG330 (Grade ≥3 in 18%; Clinicaltrial.gov identifier: NCT03224819) (72). Flotetuzumab (MGD006) is a bispecific, dual-affinity re-targeting (DART) antibody reactive with both CD3 antigen on T-cells and CD123 antigen on AML cells and MDSCs. This CD3 engaging bispecific antibody exhibited promising single agent activity in therapy-refractory AML patients with primary induction failure as well as patients with an early first relapse. CRS was observed in all AML patients treated with Flotetuzumab (73) and 58% of AML patients treated with Vibecotamab (XmAb14045), another CD3xCD123 BsAB (74). By comparison, only 10 of 46 patients (21.7%) treated with the CD3xCD123 BsAB APVO436 developed CRS (64).

IL-6 is one of the driving pro-inflammatory cytokines that contribute CRS and its pulmonary, cardiovascular, renal, and neurologic complications (60, 64–71). Cytokine profiling in patients who developed CRS after APVO436 infusion indicates that the predominant cytokine in this inflammatory cytokine response is IL-6, which agrees with our current knowledge regarding CRS that occurs in the context of BsAB therapy (20, 60, 64–71, 75). Within 1-2 days following the first dose of APVO436, the mean serum IL-6 concentration in these patients who developed CRS was elevated 145-fold over baseline (755 *vs* 5.2) and at the end of one week it was still elevated 83-fold over baseline. In most cases, CRS events were transient and medically manageable with standard of care including the use of dexamethasone and anti-IL-6:IL-6R antibody Tocilizumab or anti-IL-6 antibody Siltuximab (antibody against IL-6). However, CRS can be life-threatening even with the use of Tocilizumab or Siltuximab (60, 64–71). Therefore, development of consistently effective prevention and treatment regimens against CRS remains an urgent and unmet medical need. Identification of such regimens would further advance the field of immunotherapy. We recently reported the robust anti-inflammatory activity of RJX in animal models of CRS (76). RJX has been shown to block the production of IL-6, TNF-α, as well as TGF-β and reverse inflammation-induced tissue injury and multi-organ damage in mouse models of sepsis and CRS (76). RJX is currently being evaluated for its ability to prevent COVID-19 associated CRS in a double-blind randomized clinical study (NCT04708340). Because of its safety and easy use, RJX may emerge as an attractive adjunct to BsAB platforms to mitigate the risk of severe CRS.

## Neurotoxicity

Neurotoxicity is a treatment-emergent adverse event (AE) for BsABs, and it is often associated with CRS (77). The signs and symptoms vary from patient to patient and include headache, tremor, confusion, expressive and nominal dysphasia, impaired attention, apraxia, and lethargy occurring as early and common manifestations (77–79). Consensus grading criteria were developed by the ASTCT based on the use of the Immune Effector Cell-Associated Encephalopathy (ICE) screening tool (78). The CD19xCD3 BsAB blinatumumab has been reported to cause neurotoxicity in 70% of patients with B-lineage non-Hodgkin’s lymphomas (NHL). By comparison, it is less common with CD20xCD3 or CD123xCD3 BsABs (80). In a recent Phase 1 dose escalation study of the CD123xCD3 BsAB APVO436, APVO436-related transient neurotoxicity occurred only in 5 of 46 patients (10.9%). It occurred during the first cycle in 4 of the 5 patients and in Cycle 8 in the remaining patient. It was mild with Grade 1 AEs including headache, tremor, dizziness, lethargy, insomnia, memory loss, and confusion (64). A single case of Grade 3 confusion was encountered on the first day of treatment and resolved within a day. Neurotoxicity did not show any dose-dependence. Gender, race, age, absolute lymphocyte count or percentage of lymphocytes in peripheral blood did not predict neurotoxicity. Neurotoxicity occurred in 3 patients who also experienced CRS and in 2 patients who did not develop CRS (75). Conversely, of 10 patients who developed CRS, 7 did not experience any neurotoxicity (75).

## Conclusion

Recombinant T-cell engaging humanized BsABs redirect host T-cell cytotoxicity in an target antigen-expressing cells in patients with hematologic malignancies. They can be used both for targeting drug-resistant MM clones as well as the immune-suppressive cell populations in the BMME (**Figure 3**). Dual targeting of drug-resistant MM clones and immunosuppressive MDSC has the potential to change the therapeutic landscape for MM and improve the survival outcomes of high-risk as well as relapsed/refractory MM patients. The definition of optimal strategies for overcoming the immunosuppressive BMME in MM may require randomized clinical studies with parallel cohorts and adaptive trial designs. CD3xCD123 BsAB have clinical impact potential in MM as they may help treatment outcomes by blocking immune evasion *via* depletion of CD123^+^ MDSC and by reducing the drug-resistant tumor load *via* CTL-mediated MHC-independent destruction of MM stem cells.

## Author Contributions

FMU conceived the review, analyzed the contents of relevant publications, wrote the original draft of the manuscript revised the manuscript, provided final approval for submission of the final version. No medical writer or editor was involved.

## Conflict of Interest

FU was employed by Ares Pharmaceuticals, and he was a consultant for Aptevo Therapeutics and for Reven Pharmaceuticals.

## Publisher’s Note

All claims expressed in this article are solely those of the authors and do not necessarily represent those of their affiliated organizations, or those of the publisher, the editors and the reviewers. Any product that may be evaluated in this article, or claim that may be made by its manufacturer, is not guaranteed or endorsed by the publisher.
